# Environmental Risk Assessment as a Tool to Enhance Maritime Spatial Planning in the Mediterranean Sea: The Case Study of the Gulf of La Spezia (North-Western Italy)

**DOI:** 10.1007/s00267-026-02559-1

**Published:** 2026-08-17

**Authors:** Alessandro Sebastiani, Andrea Peirano, Mattia Barsanti, Chiara Lombardi, Luca Appolloni, Tiziana Ciuffardi, Cristian Chiavetta, Elisabetta Salvatori

**Affiliations:** 1 Italian National Agency for New Technologies, Energy and Sustainable Economic Development (ENEA), Department for Sustainability, Section Integrated and Nature-Based Solutions for Urban Regeneration (SSPT-NATURB), Rome, Italy; 2 Italian National Agency for New Technologies, Energy and Sustainable Economic Development (ENEA), Department for Sustainability, Division Anthropic and Climate Change Impacts on the Territory, Laboratory of biodiversity and ecosystem services (SSPT-IMPACT-BES), Pozzuolo di Lerici, Italy; 3 Italian National Agency for New Technologies, Energy and Sustainable Economic Development (ENEA), Department for Sustainability, Division Circular Economy, Laboratory Tools for Sustainability and Circularity (SSPT-EC-SSC), Bologna, Italy

## Abstract

Maritime Spatial Planning is a pivotal tool for the sustainable development of coastal areas and the implementation of the blue economy. It requires a spatially explicit environmental risk assessment, including risk identification, analysis and evaluation, following the principles of Ecosystem-Based Management. This study aims to test an Environmental Risk Assessment approach using the InVEST Habitat Risk Assessment model to enhance Maritime Spatial Planning in the northern Mediterranean Sea. The model requires spatial data representing the distribution of habitats and stressors, and user-provided tabular data describing their interactions; it then produces quantitative, spatially explicit risk estimates, useful to prioritize actions for risk mitigation. The approach was applied in the Gulf of La Spezia (Italy), one of the most critical areas for Maritime Spatial Planning implementation in the Northwestern Mediterranean. Indeed, the gulf is a hotspot for marine biodiversity, hosting three Marine Protected Areas with valuable habitats like the Mediterranean coral *Cladocora caespitosa*, *Posidonia oceanica* meadows, *Cymodocea nodosa* seagrass beds, and animal forests. Nonetheless, several economically important human activities are located in the area, such as shipping, shipbuilding industries, Navy bases and arsenals, aquaculture plants, fishing and diving centres, as well as ports and other nautical facilities. Results show that risk is concentrated near coastlines (within the first 250 metres from the coast) and around islands, reflecting the spatial overlap between susceptible habitats and anthropogenic activities. The major stressors are activities/infrastructures connected with nautical tourism and coastal urbanization, such as leisure boat anchorage areas, sewage pipelines, tourist ports, and nautical facilities. A set of management actions is discussed for risk reduction, including stricter local enforcement of existing regulations and the installation of eco-friendly moorings. The proposed approach has proven to be suitable to understand habitat vulnerabilities and criticalities in a Mediterranean marine test site, providing relevant evidence-based information that could eventually steer stakeholders in defining actions for risk management. Its transparency and ease of replication make it a sound choice to build ecosystem-based knowledge, which is essential for balancing socio-economic targets with the preservation of biodiversity, ecosystem functions, and ecosystem services.

## Introduction

Coastal areas provide ecosystem services (ESs) like carbon sequestration, erosion control, food production, recreation, and host a considerable number of valuable habitats and species (Liu et al. 2022; Shepard et al. 2013). ESs play a primary role for the implementation of the blue economy (Louey 2022), as they underpin critical sectors such as fisheries, tourism and blue biotechnologies (Mulazzani and Malorgio 2017; Rotter et al. 2023). However, ESs in the Mediterranean basin are at risk, since they are threatened by anthropogenic pressures, including the overexploitation of marine species, habitat loss and eutrophication (Lotze et al. 2006), and by climate change, which is causing water warming and acidification (Coll et al. 2010).

In this context, preserving biodiversity is the key not only to safeguarding ecosystem functions, which are at the basis of the ESs delivery (Buonocore et al. 2021; EC 2020), but also to unlocking the full potential of the blue economy. To achieve this, there is a broad consensus upon the adoption of the Ecosystem-based Management (EBM) approaches, defined as the “*management driven by explicit goals executed by policies, protocols and practices, and made adaptable by monitoring and research based on our best understanding of the ecological interactions and processes necessary to sustain ecosystem structure and function*” (Christensen et al. 1996). EBM is considered a best practice for managing multiple uses of maritime areas while addressing their reciprocal trade-offs (Haugen et al. 2024). The adoption of EBM in marine areas has been strongly encouraged at both the European and national levels through the Marine Strategy Framework Directive (Directive 2008/56/EC), the Water Framework Directive (Directive 2000/60/EC) and the Maritime Spatial Planning Directive (Directive 2014/89/EU).

Maritime Spatial Planning (MSP), which is the process of characterizing the spatial and temporal distribution of human activities in marine areas to achieve ecological, economic, and social objectives (Ehler and Douvere 2009), should therefore be implemented following EBM’s principles. MSP, which has advanced worldwide over the last 30 years and is now widely recognized as a pivotal element for the sustainable use of marine resources, is mandatory in several regions, including Europe (Ehler 2021); Italy adopted its Maritime Spatial Plan on 25th of September 2024 by Ministerial Decree No. 237 (EC 2024). However, integrating EBM into MSP has proven to be problematic, primarily due to the lack of standardized tools and methodologies (Galparsoro et al. 2025). In this regard, Ramieri et al. (2024) state that the future evolution of Italian Maritime Spatial Plan should rely on multi-scale, novel studies investigating land-sea interactions, landscape preservation and biodiversity conservation.

MSP should be supported by the Environmental Risk Assessment (ERA), which links human activities to ecosystem vulnerability to clarify potential conflicts (Gimpel et al. 2013). A typical ERA includes risk identification, analysis and evaluation, with outputs designed to be easily communicated to stakeholders and policy makers for the elaboration of risk’s treatment actions and strategies (Cormier et al. 2013; Stelzenmüller et al. 2015).

The objective of this study is to test an ERA using the InVEST (Integrated Valuation of Ecosystem Services and Tradeoffs) Habitat Risk Assessment (HRA) model, a free and open-source tool for assessing the risk posed to habitats by human activities (Natural Capital Project 2014). The model enables users to visualize marine areas where trade-offs among human activities and ESs occur, under current and future scenarios of maritime space use. It has been successfully applied in numerous locations across the world (e.g. Belize, Arkema et al. 2014 and Verutes et al. 2017; France, Cabral et al. 2015; South Korea, Chung et al. 2015; Brazil, Elliff et al. 2017; USA, Wyatt et al. 2017; China, Zhai et al. 2020; Portugal, Caro et al. 2020 and Bastos et al. 2023), helping to minimize the impairment of habitat quality and functions; to the best of our knowledge, it has never been applied to inform management strategies in the Mediterranean sea.

The investigation has been carried out in the Gulf of La Spezia, located in the Eastern part of the Ligurian Sea (North-Western Italy), one of the most critical areas for the European MSP Directive implementation in the Mediterranean (Fabbri et al. 2025). Although small, the gulf is an important biodiversity hotspot, hosting the Natural Regional Park of Porto Venere Marine Protected Area (MPA), the coastal Natural Regional Park of Magra-Vara-Montemarcello, and bordering the MPA of the Cinque Terre National Park. Furthermore, it is included in the Pelagos Sanctuary, the only international MPA dedicated to the protection of marine mammals and their habitats in the Mediterranean Sea (Notarbartolo di Sciara et al. 2008). Several impacting anthropogenic activities exists on this valuable natural environment, connected with the commercial and military port of La Spezia and the surrounding urban and touristic areas. Indeed, the gulf hosts Navy bases and arsenals, shipbuilding industries, cruise terminals, commercial and recreational shipping, as well as aquaculture plants, fishing and diving centres. These activities coexist and compete for the same limited maritime space, often in a poorly managed way (Vietti et al. 2010), thus offering an ideal ERA case study in a Mediterranean seascape.

The results of the present study were used to hypothesize a set of site-specific management actions whose implementation would enhance MSP under EBM principles (Micheli et al. 2013). The potential of the methodology to provide a standardized and transferable tool to support the implementation of the EU Maritime Spatial Planning Directive, the EU Biodiversity Strategy 2030, and regional frameworks such as the UNEP/MAP Barcelona Convention in the Mediterranean Sea, has been discussed.

## Materials and Methods

### Study Area

**Fig. 1:**
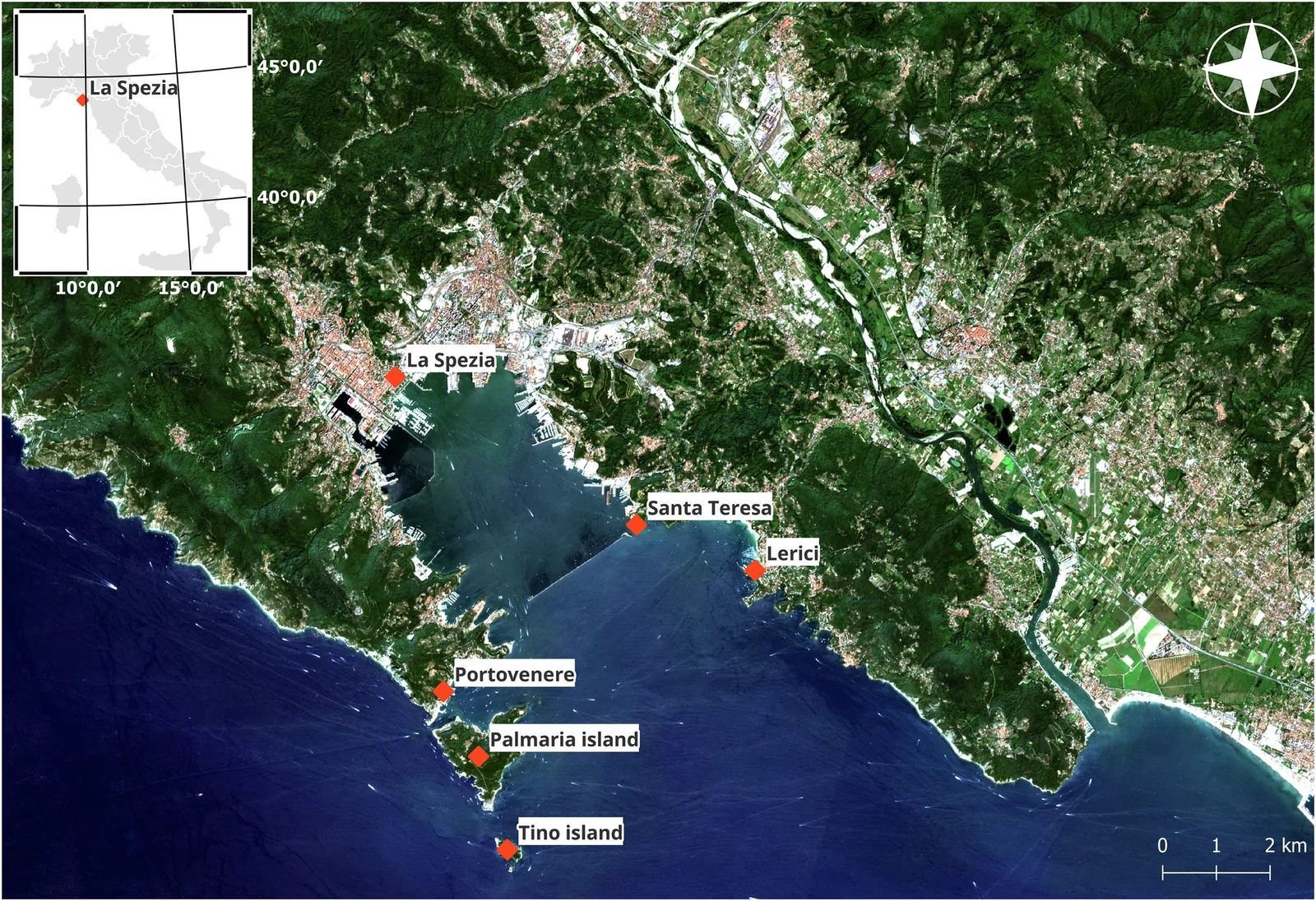
Gulf of La Spezia. Base image is a Sentinel-2 level 2A image acquired in August 2018

The Gulf of La Spezia is located along the Ligurian coast in north-western Italy (Fig. 1). It takes its name from the city of La Spezia, one of Italy’s major port cities with over 94,000 inhabitants.

The gulf is 3–4.5 km wide and with depth from 8 to 25 m; the shoreline is predominantly rocky, with steep slopes, cliffs and platforms (Rossi et al. 2021; Ciuffardi et al. 2025). To the west, it comprises the Regional Park of Porto Venere, a protected natural area that includes the islands Palmaria, Tino, and Tinetto, as well as 131 ha of surrounding MPA; this area falls within the UNESCO World Heritage Site “Portovenere, Cinque Terre and the Islands (Palmaria, Tino and Tinetto)” (UNESCO site code 826bis). To the east, it is bounded by the Regional Park of Montemarcello–Magra–Vara. Both parks have been designated as Special Areas of Conservation (IT1345005 and IT1345109, respectively). The entire area is included in the Pelagos Sanctuary for Mediterranean Marine Mammals.

The Gulf of La Spezia has been previously investigated for its habitat composition (Peirano et al. 2011, 2001), hydrodynamic conditions (Gasparini et al. 2009) and water quality (Balbi et al. 2017; Bonello et al. 2018). The gulf represents a biodiversity hotspot, hosting valuable habitats like meadows of *Posidonia oceanica* (Linnaeus) Delile, 1813, seagrass beds of *Cymodocea nodosa (Ucria) Ascherson, 1870* (Peirano and Bianchi 1997), and colonies of *Cladocora caespitosa* (Linnaeus, 1767), the only zooxanthellate coral native to the Mediterranean basin (Zibrowius 1980), which constitute one of the most important hotspots of this species in the Mediterranean Sea (Peirano et al. 2001). Another peculiarity of the area are the animal forests characterizing the coralligenous habitat. Those include species of high ecological relevance for the Mediterranean Sea, such as *Paramuricea clavata* (Risso, 1826), *Eunicella* sp., *Lophogorgia sarmentosa* (Esper, 1789), *Pentapora fascialis* (Pallas, 1766) (Bramanti et al. 2016; Lombardi et al. 2020). Along the investigated coastal sector, the macroalgal community shows a pronounced vertical zonation, as previously demonstrated in other areas of the Mediterranean Sea (de Bettignies et al. 2025). The shallowest reef belt is characterized by typical photophilous algal communities that, on natural substrates along the western side of the Palmaria island, are dominated by the algae *Halopteris scoparia* (Linnaeus) Sauvageau 1904 with the noteworthy presence of *Lithophyllum byssoides* (Lamarck) Foslie 1900 (taxonomic reference: https://www.algaebase.org) and the date mollusk *Lithophaga lithophaga*. Below 13 m or under reduced light conditions, the community becomes characterized by sciaphilous algae, such as *Halimeda tuna* (Ellis and Solander) J.V.Lamouroux 1816 (taxonomic reference: https://www.algaebase.org): this is the transitional area of the “Infralittoral sciaphilic algal assemblages” habitat, that can be referred as pre-coralligenous communities. Such biological richness is included in the annual ecological monitoring of the Porto Venere Regional Park, and its calcifying marine ecosystems are part of the Eastern Ligurian Site of the European Long-Term Ecosystem Research network (eLTER, https://elter-ri.eu/), a European research network that collects field data on climate, soil, water, and biodiversity, combining monitoring, experiments, and modelling.

Anthropogenic activities and infrastructure - including harbour activities (commercial, industrial and military), coastal urban development, tourism, and pollution discharges - have a central role in the economic development of the gulf (Vietti et al. 2010). The port of La Spezia is one of the major ports in Italy in terms of goods handled, with over 1.2 million TEU in 2024 (Port System Authority of the Eastern Ligurian Sea 2025); the port entrance is delimited by 2.2 km-long dam which separates the main port area from the open sea, significantly affecting water circulation (Ciuffardi et al. 2025).

Tourism in La Spezia and its surroundings is also highly developed, with slightly more than 1 million visitors overnight stays in 2024 (Regione Liguria 2025). Leisure boating has driven a sharp increase in the number of moorings and small harbours (Piccinno and Zanini 2010); fishing and shellfish farms are also strong blue bioeconomy sectors, the latter covering about 40 hectares of marine area, involving 91 companies and employing 114 people (Agriliguria 2010).

### InVEST Habitat Risk Assessment (HRA) Model

The InVEST HRA model is a free, open-source tool for assessing the cumulative risk posed to habitats by human activities by means of two dimensions of information, that is, exposure and consequence (Arkema et al. 2014; Natural Capital Project 2014). According to the definition provided by Bastos et al. (2023) and the Natural Capital Project (Natural Capital Project 2014), risk refers to the likelihood that anthropogenic pressures will degrade habitat quality to the point of threatening its ability to provide ESs. The model combines spatial data representing the distribution of habitat and stressors; it produces quantitative, spatial and tabular explicit estimates of their interactions based on user-defined exposure and consequence (E/C) criteria scores to estimate the risk posed to each habitat.

Exposure (E) is defined as the degree to which a habitat experiences a stressor, whereas consequence (C) refers to the habitat’s response to that stressor. Exposure criteria include spatial overlapping, overlap time rating, intensity rating and management strategy. Consequence criteria include changes in area rating, change in structure rating, frequency of natural disturbance rating, natural mortality rating, recruitment rating, recovery time and connectivity rating. These criteria are described in depth in the HRA user manual (Natural Capital Project 2014). For each stressor, a buffer distance, that is, the maximum distance at which it exerts its negative impact, must be defined. It is also required to establish a distance-decay function, that is, how the detrimental impact of each stressor decreases with distance. In this study, it was adopted a linear decay function, which assumes that stressor’s effects in the buffer area decay linearly with distance.

As in previous studies (Bastos et al. 2023; Caro et al. 2020), the E/C table was compiled through expert judgement assessment and literature data. In the present case, the expert-based evaluation involved researchers from Research Centres and Universities working in the study area, as well as local stakeholders from the Smart Bay S. Teresa project (https://smartbaysteresa.com/en/). A total of 20 experts were involved, and in particular: researchers in Marine biology (6 experts), Oceanography (3 experts), Coastal geomorphology and marine habitat mapping (2 experts), Biophysical evaluation of ecosystem services (2 experts); Associated Mussel Farmers’ Cooperative (3 experts, shellfish farmers and fisherman), NGO for marine education (3 expert) and Navy divers group (1 expert). All these experts have worked in the study area from 5 to more than 30 years. The literature data included the Report on Site-specific Conservation Measures for the Ligurian Marine Sites of Community Importance (Regione Liguria 2014). This report is the result of local studies and a large expert-based consultation and specifically addresses the relation between marine habitats and stressors at the local level. The default E/C criteria proposed by the INVEST HRA software was adopted, using a scale ranging from 1 (low exposure/consequence) to 5 (high exposure/consequence). Experts independently compiled the E/C table, then reviewed and discussed their ratings until reaching a consensus for each E/C criterion for each habitat/stressor pair. The buffer distance for each stressor was also defined using an expert-based approach, with the support of physical-biogeochemical observations, including marine current measurements, continuously acquired within the gulf by the Smart Bay Santa Teresa observatory (Ciuffardi et al. 2025). The Smart Bay Santa Teresa observatory is part of the Joint European Research Infrastructure of Costal Observatories (JERICO-RI, https://www.jerico-ri.eu/) and collects real-time data on marine currents to understand water movement; it measures the speed and direction of currents both at single points and along the entire water column. These data help track how water circulates in the area. Experts agreed that some habitats, such as *P. oceanica* meadows*, C. nodosa* beds, coralligenous outcrops, animal forests and *C. caespitosa* beds are extremely susceptible to stressors and thus received high Consequence criteria scores. Slightly lower scores were associated to habitats such as Infralittoral sciaphilic algal assemblages, that include pre-coralligenous communities which are known to be more sensitive to anthropogenic stress factors than photophilic algae (Montefalcone et al. 2017), while lower scores were attributed to *Ellisolandia elongata* and photophilic algae habitats. Conversely, habitats like coastal muds and sands were less sensitive to human pressures and received even lower scores. The complete E/C table (Supplementary Table S1) is reported in the supplementary materials.

E/C scores for each habitat/stressor pair are combined by the HRA model to calculate risk (R): habitats with high exposure and high consequence to one or more stressors will display high risk values, whereas those with low exposure and low consequence will show a low cumulative risk. The model computes the exposure and consequence for each habitat/stressor pair according to the following equations:

1 $${E}_{{jkl}}=\frac{{\sum }_{i=1}^{N}\frac{{e}_{{ijkl}}}{{d}_{{ijkl}}\cdot {w}_{{ijkl}}}}{{\sum }_{i=1}^{N}\frac{1}{{d}_{{ijkl}}\cdot {w}_{{ijkl}}}}$$

2 $${C}_{{jkl}}=\frac{{\sum }_{i=1}^{N}\frac{{c}_{{ijkl}}}{{d}_{{ijkl}}\cdot {w}_{{ijkl}}}}{{\sum }_{i=1}^{N}\frac{1}{{d}_{{ijkl}}\cdot {w}_{{ijkl}}}}$$

Where:

*E*_*jkl*_ and *C*_*jkl*_ are the exposure and consequence scores specific to habitat *j*, from stressor *k* in cell *l*; *e*_*ijkl*_ and *c*_*ijkl*_ are the exposure and consequence rating criterion *i*, specific to habitat *j* and stressor *k* and cell *l*; *d*_*ijkl*_ is the data quality rating, while *w*_*ijkl*_ is the importance weighting for the criterion. *N* is the number of criteria for each habitat. For the present study, data quality was set to the highest possible score for each habitat-stressor pair, whereas the importance weighting was set to 1, indicating equal relative importance of the E/C criteria in determining the risk.

Exposure and consequence are then combined to calculate the risk. Euclidean risk calculation, which was adopted in this study, is expressed as:

3 $${R}_{{jkl}}=\sqrt{{\left({E}_{{jkl}}-1\right)}^{2}+{\left({C}_{{jkl}}-1\right)}^{2}}\cdot {D}_{{jkl}}$$

Where *R*_*jkl*_ is the risk to habitat *j* caused by stressor *k* in each cell*l*; *D*_*jkl*_ is the distance-weighted decay, which is linear in the present study. Cumulative risk for habitat *j* from all stressors in cell *l* is the sum of all risk scores for that habitat (Eq. (4)):

4 $${R}_{{jl}}=\mathop{\sum }\limits_{k=1}^{K}{R}_{{jkl}}$$

Eventually the cumulative risk, which is an integrative index of risk across all habitats in a grid cell, is computed by summing habitat risk scores in each cell (Eq. (5)):

5 $${R}_{l}=\mathop{\sum }\limits_{J=1}^{J}{R}_{{jl}}$$

Each cell is then classified as none, low, medium, or high-risk using Eq. (6):

6 $$6)\,{LEl}=\,\left\{\begin{array}{lll}\begin{array}{cc}0\,{if} & {Rl}=0\\ 1\,{if}\, & 0 < {Rl} < \,\frac{1}{3}{ql}\\ 2\,{if}\, & \frac{1}{3}{ql}\le {Rl} < \,\frac{2}{3}{ql}\,\end{array}\\ \begin{array}{cc}3\,{if}\, & \,{Rl}\ge \,\frac{2}{3}{ql}\,\end{array}\end{array}\right\}$$

where $L{E}_{l}$ represents the risk class at cell *l* (equation nit shown), ${R}_{l}$ is the cumulative risk across all habitats at that location;${q}_{l}$ is the maximum possible risk score, calculated as ${q}_{l}={m}_{{jkl}}\times {n}_{j}$, where ${n}_{j}$ is the total number of habitats provided by the user. The analysis resolution was set to 20 m.

Results are delivered in both raster and tabular formats. Raster outputs include the ecosystem risk map, which is the sum of habitat cumulative risk scores divided by the number of habitats occurring in each cell, and the risk classification map, which divides the study area into high (R_%HIGH), medium (R_%MEDIUM), low (R_%LOW) and no (R_%NONE) risk zones. Grid cells were classified as no risk when risk scores were equal to 0, as low risk when risk score is below 33% of the maximum possible risk, as moderate risk when risk score ranges between 33% and 66% of the maximum possible risk, and as high risk when risk score exceeds 66% of the maximum possible risk (Eq. (6); Bastos et al. 2023). It is worth reminding that, within the InVEST HRA framework, no-risk zones are those where there is no spatial overlap between habitats and stressors. Tabular data include several Exposure, Consequence and risk metrics, comprising mean habitat exposure (E_MEAN), mean habitat consequence (C_MEAN), maximum and mean habitat risk (R_MAX and R_MEAN, respectively), as well as R_%HIGH, R_%MEDIUM, R_%LOW and R_%NONE expressed as the share of the total area of each habitat. All the metrics are reported at the habitat–stressor pair level and for all stressors combined. For further details on the HRA model, please refer to the user manual (Natural Capital Project 2014).

### Geospatial Data Collection

**Fig. 2:**
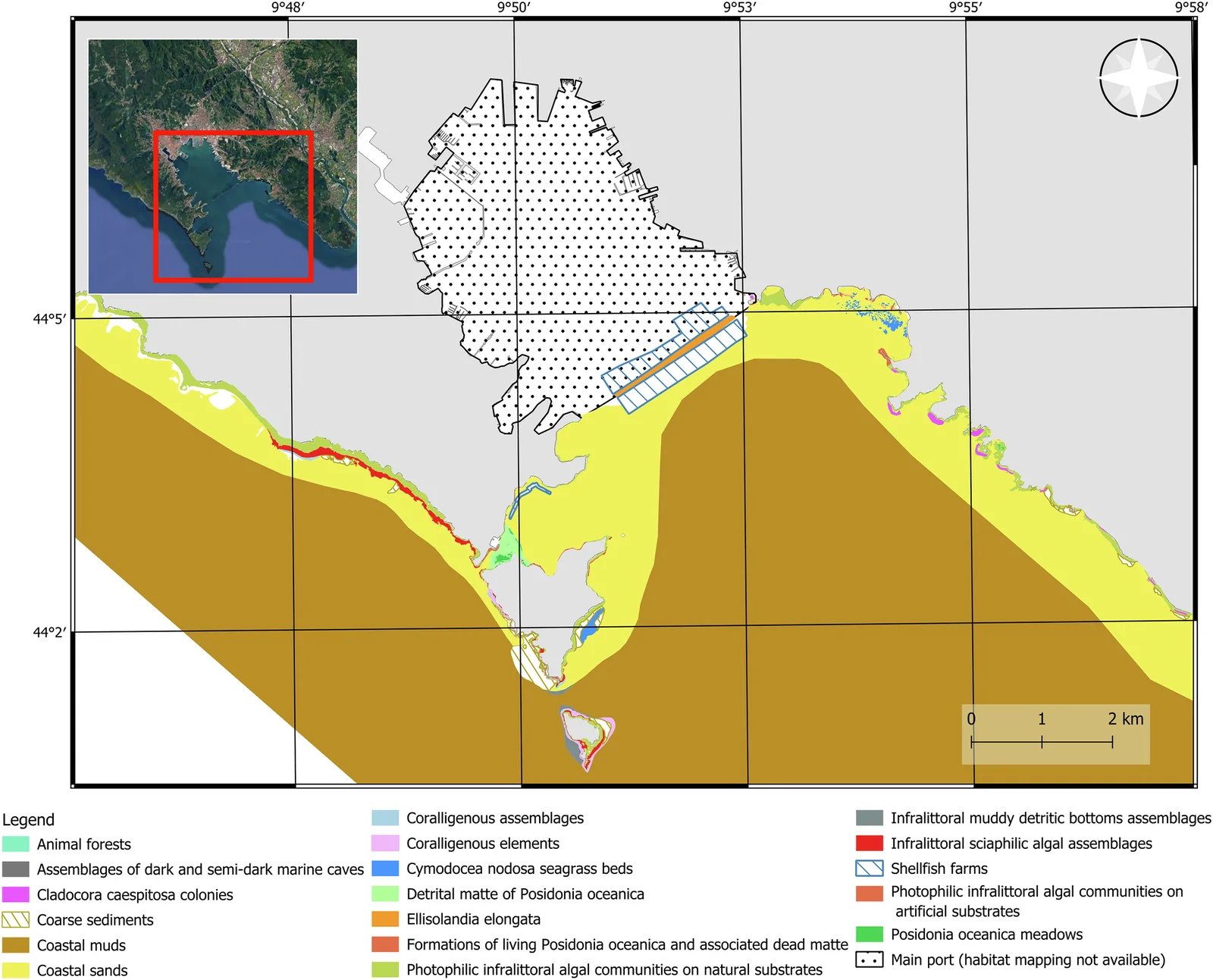
Spatial distribution of main benthic marine habitats of the Gulf of La Spezia

Geospatial data concerning habitats and stressors were gathered from multiple local sources. A total of 18 habitats were considered (Table 1). The main data source was the Marine habitat Atlas of Liguria Region 2020 (MHA2020), available from the Ligurian regional Geoportal (Regione Liguria 2020). For certain habitats (see Table 1), MHA2020 data were combined with field sampling conducted by the ENEA marine Environment Research Centre Santa Teresa. These surveys identified additional habitats not reported in MHA2020, such as animal forests and fringe of *E. elongata* (Ellis and Solander) (Hind and Saunders 2013; Nannini et al. 2015; Marchini et al. 2019; Ragazzola et al. 2021). In the case of overlapping information between the MHA2020 and field sampling campaigns, priority was given to the latter (Fig. 2).

**Table 1:** Habitat name, data source, dominant substrate and total surface area occupied in the study area

Habitat name (acronym)	Source	Substrate	Total area (ha)
Shellfish farms (shf)	MHA2020 and field studies	Sandy	109.5
*Cymodocea nodosa* seagrass beds (cn)	MHA2020 and field studies	Sandy	13.1
*Posidonia oceanica* meadows (pom)	MHA2020 and field studies	Sandy	2.9
Formations of living *Posidonia oceanica* and associated dead matte (podm)	MHA2020	Sandy	0.1
Detrital matte of *Posidonia oceanica* (dmpo)	MHA2020	Sandy	13.5
Coastal sands (cs)	MHA2020	Sandy	1108.5
*Ellisolandia elongata* (ee)	field studies	Rocky	17.8
Photophilic infralittoral algal communities on artificial substrates (paa)	MHA2020	Rocky	5.4
Infralittoral sciaphilic algal assemblages (isaa)	MHA2020	Rocky	13.9
Photophilic infralittoral algal communities on natural substrates (pan)	MHA2020	Rocky	176.2
*Cladocora caespitosa* colonies (ccc)	MHA2020 and field studies	Rocky	17.2
Coralligenous elements (ce)	MHA2020	Rocky	9.3
Assemblages of dark and semi-dark marine caves (adsdmc)	MHA2020	Rocky	0.1
Coralligenous assemblages (ca)	MHA2020	Rocky	4.0
Animal forests (af)	field studies	Rocky	0.1
Coarse sediments (cse)	MHA2020	Sandy	8.2
Infralittoral muddy detritic bottoms assemblages (imdb)	MHA2020	Muddy	8.2
Coastal muds (cm)	MHA2020	Muddy	4594.3

For the data sources, MHA2020 is the Marine habitat Atlas of Liguria Region 2020

**Fig. 3:**
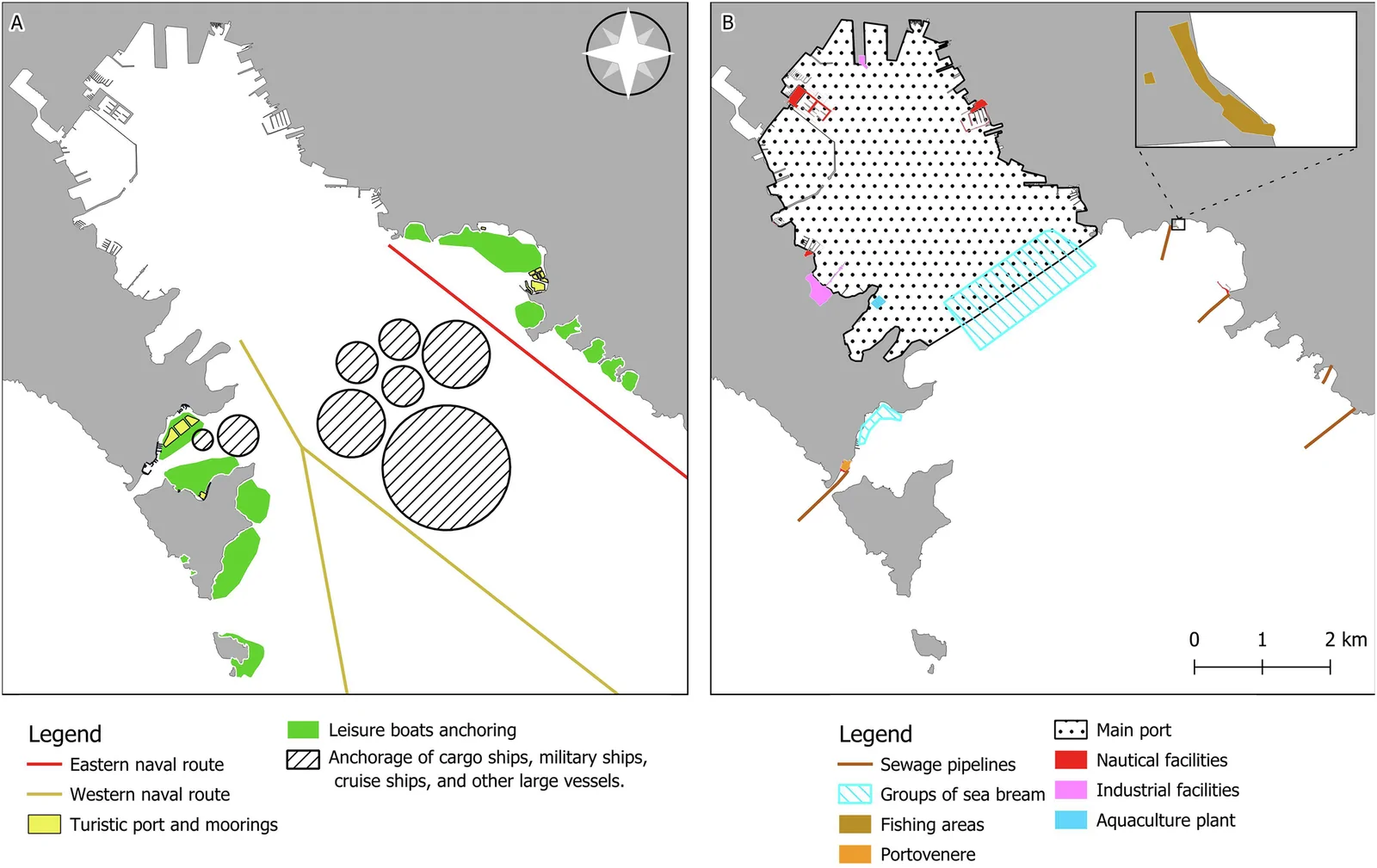
Main marine stressors of the Gulf of La Spezia. Panel **A** reports stressors classified as Navigation-related; Panel **B** reports Anthropogenic and Biological stressors and infrastructure

Stressors were mapped using both literature and field data (Fig. 3). These have been categorized in: Anthropogenic activities, Navigation, Infrastructure, and biological stressors. Overall, the portfolio of stressors is well representative of the study area. Most stressor layers were downloaded from the Ligurian regional geoportal (LRG), complemented by field campaigns and maps from the Hydrographic Institute of the Italian Navy (HIN). Interestingly, there is also a biological stressor represented by sea breams. Over the last years, sea breams in the study areas have supposedly increased their numbers and voracity (no peer-reviewed study has yet been published on this topic), causing a steep decrease in the shellfish production via predation. Data sources are listed in detail in Table 2.

**Table 2:** Stressors in the study area

Stressor	Category	Source
Fishing areas	Anthropogenic activity	LRG
Aquaculture plant	Anthropogenic activity	LRG
Leisure boats anchoring sites	Navigation	Field campaigns
Anchorage of cargo ships, military ships, cruise ships, and other large vessels.	Navigation	HIN (table n. 60 INT3365)
Western naval route	Navigation	HIN (table n. 60 INT3365)
Eastern naval route	Navigation	HIN (table n. 60 INT3365)
Groups of sea bream	Biological	Field campaigns
Nautical facilities	Infrastructure	LRG
Sewage pipelines	Infrastructure	LRG
Touristic port and moorings	Navigation	LRG
Industrial facilities	Infrastructure	LRG
Main port	Infrastructure, Anthropic activities	LRG
Porto Venere	Infrastructure	LRG

LRG is the Ligurian regional geoportal; HIN is the Hydrographic Institute of the Italian Navy; field campaigns do not refer to any specific campaign

Each of the stressors might impact habitats in multiple ways, including chemical pollution, noise pollution, physical or biological damage to the seafloor. Leisure boating anchoring sites are known to have a detrimental impact on the marine environment (Carreño and Lloret 2021), with *P. oceanica* and *C. nodosa* meadows being severely jeopardised by anchoring; several illegal leisure boat anchoring sites are located inside or in proximity of the MPA of Porto Venere. The “main port” is a layer for multiple pressures generated by the La Spezia port, including military operations, shipyard activities, dredging and noise. The finfish aquaculture plant located inside the main port also contributes to water pollution. Sea bream predation on shellfish farms drastically reduces yields (Glamuzina et al. 2014), and local farmers have reported significant losses due to predation (Regione Liguria 2023). The western naval route is navigated by oil tankers, cruise vessels, and fishing boats, and it is more impactful compared to the eastern route, which is instead navigated by much smaller touristic boats. Larger vessels often anchor in designated areas outside the port, causing seafloor excavation and physical disturbance (Watson et al. 2022). Sewage pipelines discharge pollutants and nutrients, leading to oxygen depletion and plant life decay (Vikas and Dwarakish 2015). Porto Venere’s leisure marine traffic, docks, and moorings also generate chemical and noise pollution and physical damage to the seabed.

### Analysis of the Main Model Outputs

Spatial and tabular model’s outputs were utilized to analyse the spatial distribution of risk across the study area. A buffer analysis was performed to assess the distribution of the different risk classes at different distances from the coast. Three buffers were defined: 0–250 m, 250–500 m, and 0–500 m. Within this buffer, the abundance of risk classes was computed, as both the percentage of the total area of each risk class occurring within the buffer (C%) and the percentage of the buffer area occupied by each risk class (B%). Subsequently, tabular outputs produced by the model were used to identify habitats at higher risk and stressors exerting greater pressure, and to assess habitat-stressor pairs at higher risk. The mean risk score for each habitat–stressor pair (computed following Eq. (3)) is a measure of the pressure posed to each habitat by every single stressor. Priority habitat–stressor pairs were identified using the mean risk as the selection criterion: of the 234 habitat-stressor pairs, those exceeding the 80th percentile per mean risk were selected and included in the management scenario.

### Management Scenario

Potential actions aimed at contrasting the risk in critical areas were hypothesized and included in a management scenario, which was eventually used to re-run the InVEST HRA model. In the considered scenario, all E/C scores (excluding leisure boat anchoring sites, which will be addressed later in this section) were left unmodified except for the criteria “management effectiveness”, whose score was instead set to 1, which means that management was made extremely effective. Since the management criteria refers to exposure, lowering its value simulates a lower exposure of the selected habitat to that stressor. Therefore, proposed actions should aim at drastically reducing the impact of stressors, mostly through the adoption low-impact practices. Leisure boat anchoring sites were completely removed from the stressor portfolio, as anchoring is already explicitly prohibited in these areas, although local enforcement is still not fully effective. Therefore, the management scenario assumes a strengthening of the current legislation and its enforcement regarding leisure boat anchoring.

## Results

### Ecosystem Risk

**Fig. 4:**
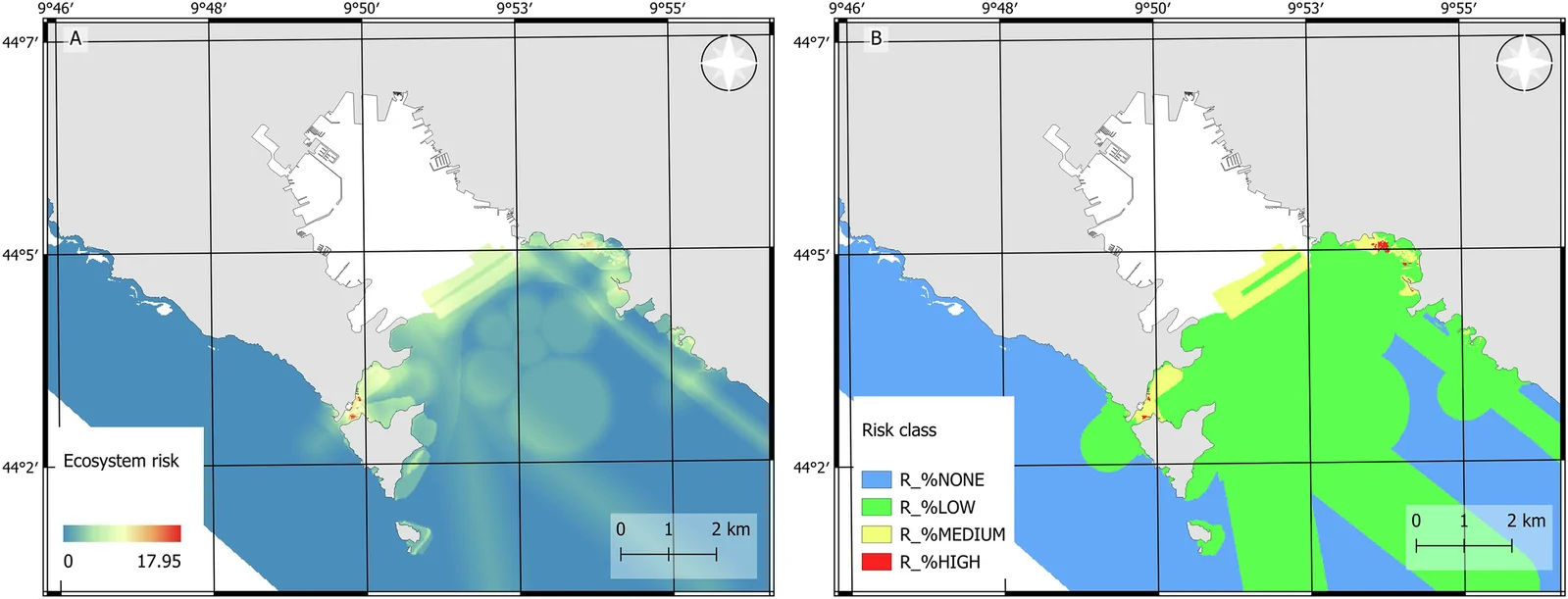
Panel **A**: Ecosystem Risk (R) map of the Gulf of La Spezia; Low-risk values are shown in shades of blue; medium-risk values in light yellow shades; and high-risk values in shades of red. Panel **B**: Risk classification map; The colours blue, green, yellow, and red represent the no-risk (R_&NONE), low-risk (R_%LOW), medium-risk (R_%MEDIUM), and high-risk (R_%HIGH) zones, respectively

Most of the area is at low to no risk, with higher-risk zones being highly localized where multiple stressors insist on vulnerable habitats. In particular, 6.3 ha were classified at high risk; 207.2 ha at medium risk; 3822.7 ha at low risk, and 5302.2 ha at no risk. Higher risk is mostly found close to the coastline (in the 0–250 m buffer, Table 3) in the eastern part of the study area, and between Porto Venere and Palmaria island on its western borders. Shellfish farms right outside the main port shows moderate risk levels; overall, even though there are plenty of stressors like naval routes and anchorage sites, open sea areas are populated by muddy and sandy habitats, which have low consequence scores, thus resulting in low-risk values (Fig. 4A, B, Table 3).

**Table 3:** Buffer analysis showing the distribution of environmental risk classes as a function of distance from the coastline

Buffer distance from the coastline	R_%HIGH			R_%MEDIUM			R_%LOW			R_%NONE		
	**ha**	**C%**	**B%**	**ha**	**C%**	**B%**	**ha**	**C%**	**B%**	**ha**	**C%**	**B%**
0–250 m	3.8	60.5	0.6	71.2	34.4	11.0	434.6	11.3	66.9	139.8	2.6	21.5
250–500 m	0.3	4.8	0.1	19.2	9.3	3.5	376.6	9.9	69.1	149.3	2.8	27.4
0–500 m	4.1	65.3	0.3	90.4	43.6	7.6	811.2	21.2	67.9	289.1	5.5	24.2

For each buffer, the table reports: the area (ha) of each risk class, the percentage of the total area of each risk class occurring within the buffer (C%), and the percentage of the buffer area occupied by each risk class (B%)

It’s important to note that, given the presence of abrupt changes in shoreline orientation, local deviations from the nominal buffer distance (250 m) occur in the study area; for this reason, the 0–250 m buffer is roughly 100 hectares larger than the 250–500 m, which may cause misinterpretation of the results.

**Fig. 5:**
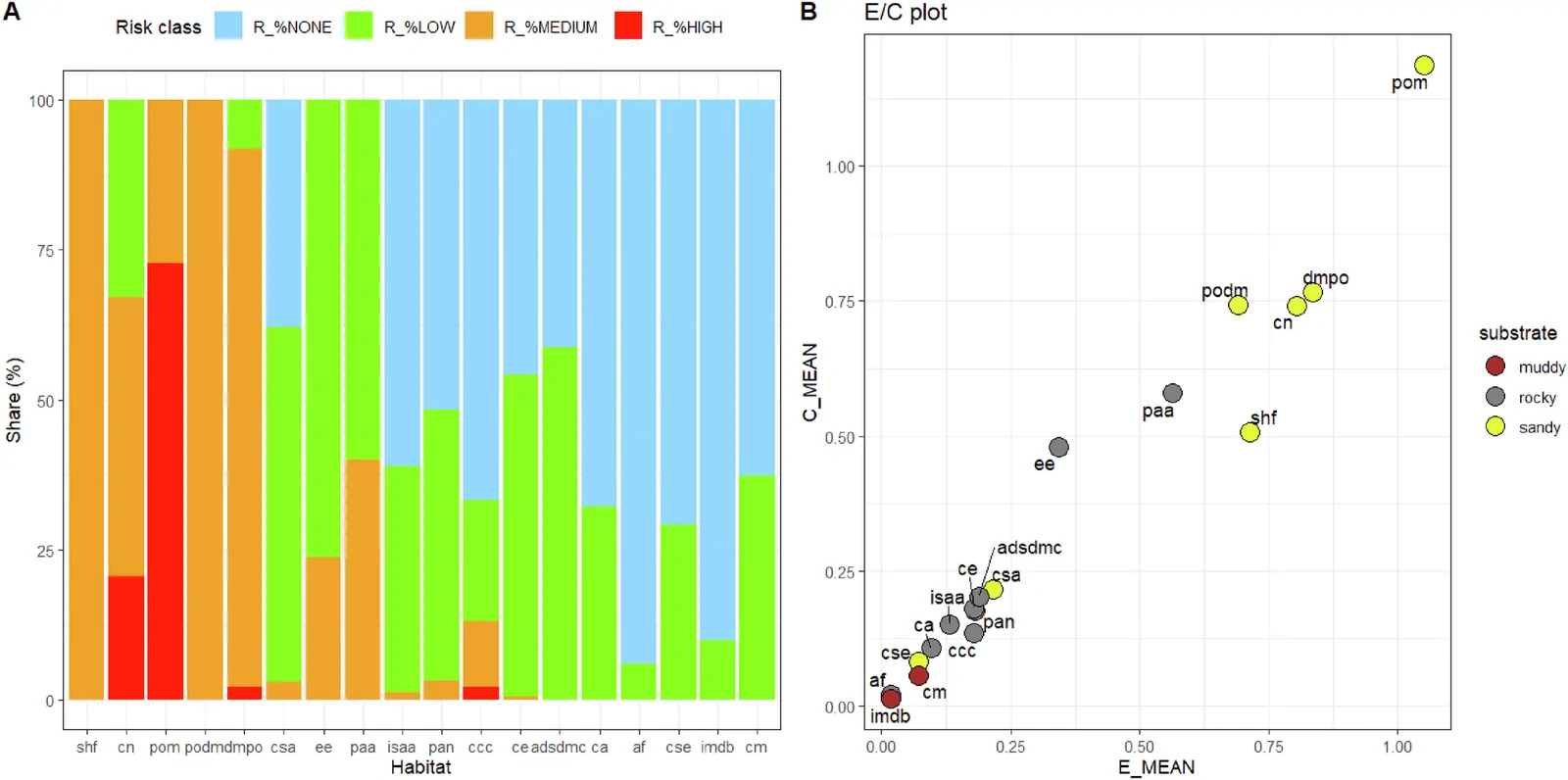
Panel **A**: Stacked bar chart reporting the proportion of each habitat’s area per risk class. Panel **B**: Scatter plot reporting mean consequence on the y axis and mean exposure on the x axis. Habitat acronyms: Shellfish farms (shf); *Cymodocea nodosa* seagrass beds (cn); *Posidonia oceanica* meadows (pom); Formations of living *Posidonia oceanica* and associated dead matte (podm); Detrital matte of *Posidonia oceanica* (dmpo); Coastal sands (cs); *Ellisolandia elongata* (ee); Photophilic infralittoral algal communities on artificial substrates (paa); Photophilic infralittoral algal communities on natural substrates (pan); *Cladocora caespitosa* colonies (ccc); Coralligenous elements (ce); Assemblages of dark and semi-dark marine caves (adsdmc); Coralligenous assemblages (ca); Animal forests (af); Coarse sediments (cse); Infralittoral muddy detritic bottoms assemblages (imdb); Coastal muds (cm)

*P. oceanica* meadows are the habitat showing the highest risk, with 72.8% of the total habitat area being classified at high risk and 27.2% at medium risk (Fig. 5A, Table 4). *C. nodosa* beds are also highly threatened, with 20.6% of its total habitat area falling in the high-risk class and 46.5% in the medium-risk class. Accordingly, formations of living *P. oceanica* and *C. nodosa* beds have the highest and the third-highest mean risk score (1.11 and 0.67, respectively), while detrital matter of *P. oceanica* ranks second (0.80). As for *Cladocora* beds, 2.22% of these fall within the high-risk class, 10.94% in the medium and 20.22% in the low-risk class, with a mean risk score of 0.14. Shellfish farms fall entirely in the medium risk class, with a mean risk of 0.59. Habitats like coastal sands and coastal muds fall almost entirely in the low or none-risk class, and show low mean risk values (Fig. 5A, Table 4). Several sandy habitats suffer from high mean Exposure and Consequence and are therefore at risk. Most rocky habitats show moderate to low risk levels, whereas for muddy habitats are risk is extremely low or absent (Fig. 5B).

**Table 4:** Mean exposure (E_MEAN), mean consequence (C_MEAN), maximum risk (R_MAX) and mean risk (R_MEAN) scores

Habitat	E_MEAN	C_MEAN	R_MAX	R_MEAN	R_%HIGH	R_%MEDIUM	R_%LOW	R_%NONE
*Posidonia oceanica* meadows	1.05	1.19	5.66	1.11	72.83	27.17	0.00	0.00
*Formations of living Posidonia oceanica and associated dead matte*	0.69	0.74	5.66	0.80	0.00	100.00	0.00	0.00
*Cymodocea nodosa* seagrass beds	0.80	0.74	4.58	0.67	20.62	46.51	32.87	0.00
Detrital matte of *Posidonia oceanica*	0.84	0.77	3.17	0.61	2.16	89.66	8.19	0.00
Shellfish farms	0.71	0.51	3.77	0.59	0.00	100.00	0.00	0.00
*Ellisolandia elongata*	0.34	0.48	3.10	0.42	0.00	23.73	76.27	0.00
Photophilic infralittoral algal communities on artificial substrates	0.56	0.58	3.69	0.40	0.00	40.05	59.95	0.00
Coralligenous elements	0.18	0.18	4.89	0.15	0.00	0.61	53.54	45.86
Assemblages of dark and semi-dark marine caves	0.19	0.20	5.21	0.14	0.00	0.00	58.82	41.18
*Cladocora caespitosa* colonies	0.18	0.14	4.63	0.14	2.22	10.94	20.22	66.62
Coastal sands	0.22	0.22	2.20	0.12	0.00	3.12	59.03	37.86
Photophilic infralittoral algal communities on natural substrates	0.18	0.18	3.69	0.12	0.00	3.16	45.24	51.61
Coralligenous assemblages	0.10	0.11	5.21	0.09	0.00	0.00	32.19	67.81
Infralittoral sciaphilic algal assemblages	0.13	0.15	3.37	0.08	0.00	1.27	37.69	61.04
Coarse sediments	0.07	0.08	2.83	0.05	0.00	0.05	29.18	70.77
Coastal muds	0.07	0.06	1.59	0.03	0.00	0.00	37.42	62.58
Animal forests	0.02	0.02	2.56	0.01	0.00	0.00	6.12	93.88
Infralittoral muddy detritic bottoms assemblages	0.02	0.01	1.59	0.01	0.00	0.00	10.03	89.97

Columns R_%HIGH, R_%MEDIUM, R_%LOW and R_%NONE represent the share of the area of each habitat in each of the four risk classes (high, medium, low and none)

### Major Habitat/Stressor Pairs

Across 234 habitat/stressor pairs, thirteen had a mean risk value exceeding 2.0, with two of these surpassing 5.0. A total of 125 habitat/stressor pairs have a mean risk score of zero, meaning that they do not interact in any way. In total, 23 habitat/stressor pairs exceeding the 80th percentile per mean risk were selected, including 10 different habitats and 7 stressors. Sewage pipelines constitute the most recurrent stressor, reported in seven habitat–stressor combinations, consistently associated with moderate to high exposure (E_MEAN 1.13–4.70) and consequence values (C_MEAN 0.81–4.70). Leisure boats anchoring sites, the second most common stressor (five occurrences), show relatively high exposure (E_MEAN 1.28–4.30) and consequence scores (C_MEAN 0.82–5.00), particularly where they overlap with *P. oceanica* habitats. Touristic ports and moorings, occurring four times, display intermediate levels of exposure (E_MEAN 1.94–2.60) and consequence (C_MEAN 2.25–3.00). Less frequent stressors, such as nautical facilities, main port, and Porto Venere (each appearing twice), exhibit low to moderate exposure and consequence values for habitats, with E_MEAN in the range of 1.98–4.10 and C_MEAN in the range of 1.32–2.40. The least represented stressor, groups of sea bream, is associated with shellfish farms, showing moderate exposure (E_MEAN 4.00) and consequence values (C_MEAN 3.30) (Table 5).

**Table 5:** Top 80th percentile habitat/stressor pairs per mean risk score

Habitat	Stressor	E_MEAN	C_MEAN	R_MEAN
Formations of living *Posidonia oceanica* and associated dead matte	Leisure boats anchoring sites	4.30	5.00	5.20
Formations of living *Posidonia oceanica* and associated dead matte	Sewage pipelines	4.70	4.70	5.20
*Posidonia oceanica* meadows	Sewage pipelines	4.10	4.10	4.40
*Cymodocea nodosa* seagrass beds	Leisure boats anchoring sites	4.00	3.90	4.20
Shellfish farms	Groups of sea bream	4.00	3.30	3.80
Shellfish farms	Main port	4.10	2.40	3.40
*Ellisolandia elongata*	Main port	3.70	2.60	3.10
*Posidonia oceanica* meadows	Touristic port and moorings	2.60	3.00	3.00
*Posidonia oceanica* meadows	Leisure boats anchoring sites	2.10	2.50	2.50
Detrital matte of *Posidonia oceanica*	Touristic port and moorings	2.30	3.00	2.40
*Cymodocea nodosa* seagrass beds	Touristic port and moorings	2.40	2.30	2.20
*Posidonia oceanica* meadows	Nautical facilities	2.20	2.20	2.10
Detrital matte of *Posidonia oceanica*	Sewage pipelines	2.80	2.10	2.10
*Posidonia oceanica* meadows	Porto Venere	1.98	2.06	1.86
Photophilic infralittoral algal communities on artificial substrates	Touristic port and moorings	1.94	2.25	1.60
Photophilic infralittoral algal communities on artificial substrates	Sewage pipelines	2.13	1.67	1.56
Coralligenous elements	Leisure boats anchoring sites	1.37	1.44	1.43
Detrital matte of *Posidonia oceanica*	Nautical facilities	2.05	1.32	1.20
*Cymodocea nodosa* seagrass beds	Sewage pipelines	1.66	1.37	1.16
Detrital matte of *Posidonia oceanica*	Porto Venere	1.78	1.53	1.06
Assemblages of dark and semi-dark marine caves	Sewage pipelines	1.26	1.26	1.03
Detrital matte of *Posidonia oceanica*	Leisure boats anchoring sites	1.28	0.82	0.91
*Cladocora caespitosa* colonies	Sewage pipelines	1.13	0.81	0.89

Table shows mean exposure (E_MEAN), consequence (C_MEAN), and risk (R_MEAN)

### Management Actions and Expected Effects

Based on the results reported in Table 5, a set of specific management measures is proposed to reduce habitats exposure to the identified stressors; in some cases (e.g. sea breams and leisure boat anchoring sites) these have already been identified and tested by local authorities. For anchoring sites, docks and other nautical facilities the most suitable solution involves adopting seagrass friendly installations to minimize anchoring impact. For instance, to limit seagrass abrasion, Luff et al. (2019) attached small floats on a mooring chain and observed that after three years seagrass density was twice as high as that observed around traditional mooring chains.

In the case of the illegal leisure boats anchoring, stricter enforcement of the existing prohibition measures should be sufficient to completely remove the pressure. This may include stricter controls by the Port authority, especially on the most vulnerable sites, and a more efficient system of sanctions. For the sea breams, there is a management plan aimed at controlling the number of individuals through selective fishing. Light deterrent systems, such as the use of diffuse light during night, are also being evaluated. Other solutions, like the use of ultrasounds, have proven to be ineffective over the long term.

Regarding sewage pipelines, literature concerning risk management and reduction is quite scarce, and most of the papers refers to the construction and maintenance phases. Effective management of coastal sewage should integrate advanced treatment technologies, clear regulatory frameworks, and systematic monitoring (Rangel-Buitrago et al. 2024). Finally, the port areas should undergo pollution reduction measures as well as the adoption of seagrass friendly installations. Emenike et al. (2022) suggested to apply wastewater treatments such as phytoremediation and bio-absorption to metal polluted sites.

**Fig. 6:**
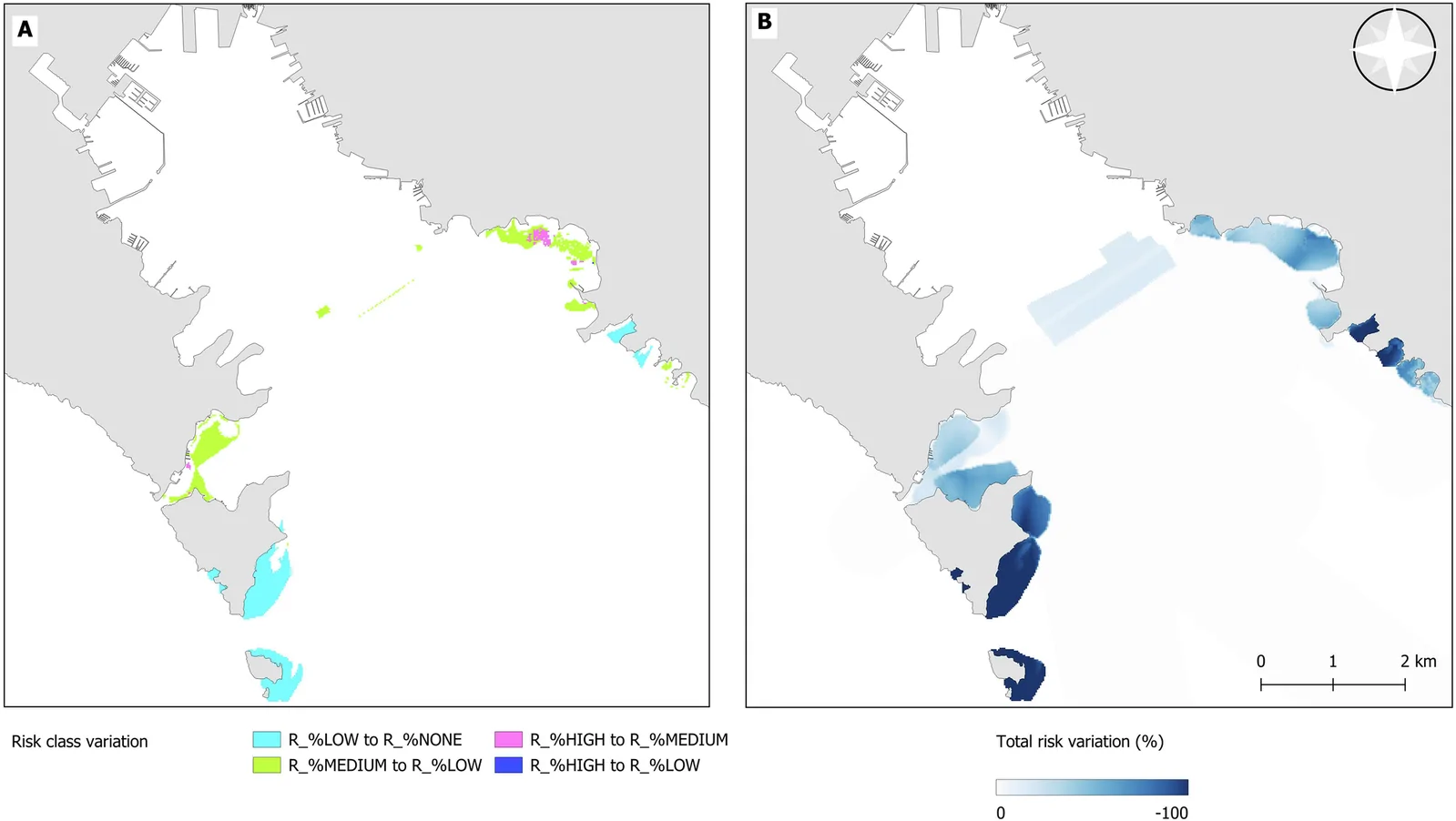
Ecosystem risk variation following the proposed management scenario. Panel **A** shows the risk reclassification occurred between and after the management scenario, panel **B** shows the ecosystem risk variation expressed in percentage

Overall, as a result of the proposed management scenario, 76.84 hectares transitioned from the low-risk to the no-risk category; 63.28 hectares moved from medium-risk to low-risk; 5.44 hectares shifted from high-risk to medium-risk; and 0.040 hectares changed from high-risk to low-risk (Fig. 6A).

Mean risk decreases in the range of 7.7–73.4% across habitats included in the management scenario (Table 6). *P. oceanica* meadows have a mean risk reduction of 25.7%, while the area classified at high risk declined from 72.8% to 46.2%, with most reclassifications shifting to the low-risk category. Other *P. oceanica* formations (e.g. detrital matte and *P. oceanica* and associated dead matte) also benefited from the proposed management. For *C. nodosa* beds, mean risk decreased by 53.8%, with high-risk areas shrinking by 19.8%. *C. caespitosa* colonies showed a 38.0% risk reduction, while *E. elongata* experienced a smaller but notable decrease (2.7%), with 9.8% of its area moving from medium to low risk. The medium-risk class consistently decreased across all these habitats, while low-risk areas expanded. Shellfish farms showed negligible improvements, remaining entirely in the medium risk class.

**Table 6:** Mean risk (R_MEAN), and share of the habitat area classified as high, medium, low and no risk (R_%HIGH, R_%MEDIUM, R_%LOW and R_%NONE, respectively) in the proposed management scenario

Habitat	R_MEAN	R_%HIGH	R_%MEDIUM	R_%LOW	R_%NONE	∆ R_MEAN (%)	∆ R_%HIGH	∆ R_%MEDIUM	∆ R_%LOW	∆ R_%NONE
*Posidonia oceanica* meadows	0.8	46.2	26.6	27.2	0.0	−25.7	−26.6	−0.6	+27.2	0.0
Detrital matte of *Posidonia oceanica*	0.5	0.0	47.8	52.2	0.0	−24.1	−2.2	−41.8	+44.0	0.0
Shellfish farms	0.5	0.0	99.2	0.8	0.0	−7.7	0.0	−0.8	+0.8	0.0
*Ellisolandia elongata*	0.4	0.0	13.9	86.1	0.0	−2.7	0.0	−9.8	+9.8	0.0
*Formations of living Posidonia oceanica and associated dead matte*	0.4	0.0	0.0	100.0	0.0	−54.3	0.0	−100.0	+100.0	0.0
*Cymodocea nodosa* seagrass beds	0.3	0.8	22.0	66.5	10.7	−53.8	−19.8	−24.5	+33.6	+10.7
Photophilic infralittoral algal communities on artificial substrates	0.3	0.0	29.9	70.1	0.0	−17.0	0.0	−10.2	+10.2	0.0
*Cladocora caespitosa* colonies	0.1	0.1	2.1	29.9	67.9	−38.0	−2.1	−8.9	+9.7	+1.2
Assemblages of dark and semi-dark marine caves	0.1	0.0	0.0	52.9	47.1	−21.1	0.0	0.0	-5.9	+5.9
Coralligenous elements	0.0	0.0	0.6	29.7	69.7	−73.4	0.0	0.0	−23.8	+23.8

Last five columns (∆ R mean, ∆ R_%HIGH, ∆ R_%LOW, ∆ R_%MEDIUM, ∆ R_%NONE) indicate the variation of the mean risk and of the total area of each habitat assigned to the different risk classes before and after the management

High-risk areas declined sharply in the first 250 m buffer from the coastline (from 3.8 to 0.8 hectares, equal to −78.9%). A similar trend was observed for the medium-risk class, which decreased by 53.3% within the same buffer area. The low-risk class had negligible changes within the first 500 m buffer. Conversely, the no-risk class increased in each of the considered buffer areas. Its increase was higher in the first 250 m buffer (+49.6%), and substantially lower (+29.5%), but still remarkable, in the 250–500 m buffer (Table 7).

**Table 7:** Buffer analysis showing the distribution of risk classes by distance from the coastline under the management scenario

Buffer distance from the coastline	R_%HIGH		R_%MEDIUM		R_%LOW		R_%NONE	
	**ha**	**∆ (%)**	**ha**	**∆ (%)**	**ha**	**∆ (%)**	**ha**	**∆ (%)**
0–250 m	0.8	−78.9	33.28	−53.3	416.4	−4.2	209.2	+49.6
250–500 m	0	−100.0	10.24	−46.7	373.4	−0.8	193.4	+29.5
0–500 m	0.8	−80.5	43.52	−51.9	789.8	−2.6	402.6	+39.3

The ∆ (%) column indicates the change in the extent of each risk class compared to current conditions

## Discussion

### Risk Distribution Across Space and Habitats in the Case Study

Coastal and marine policy frameworks are increasingly mandating a transition to an EBM to reconcile economic and social targets with the preservation of vital ecosystem services. Unlike traditional, single-sector, or habitat-specific management, EBM offers a holistic, integrated and adaptive approach that considers the entire ecosystem—including humans—to maintain health, resilience, and productivity (EC 2021). This is particularly important in the Mediterranean Sea, which serves as a critical biodiversity hotspot while simultaneously being one of the world’s most heavily impacted marine regions, due to intense, often conflicting, anthropogenic activities (Coll et al. 2010).

The results of this study, carried out in the Gulf of La Spezia, a representative test site in the Northwestern Mediterranean, support the hypothesis that risk is concentrated nearshore, within the first 250 metres from the coast, where anthropogenic pressures such as anchoring sites, ports, and sewage pipelines prevail the most.

Leisure boats anchoring sites, sewage pipelines, nautical facilities and ports substantially contribute to jeopardise highly valuable seagrasses such as *P. oceanica* (including living meadows and dead matte formations) and *C. nodosa* beds, primarily through direct mechanical abrasion (Demers et al. 2013) and sewage outfalls (Peirano and Bianchi 1997). The results are consistent with those of Catucci et al. (2019), who used a Machine Learning approach, identified variables such as distance from ports, distance from the coast, and seawater pollutant load as relevant predictors of *P. oceanica* distribution and vulnerability patterns. In this context, distance from the coast should be interpreted as a spatial proxy related to environmental gradients (e.g., depth and turbidity), rather than as a direct driver of vulnerability. This interpretation is also consistent with findings of Tuya et al. (2014), who identified proximity to human infrastructures as a strong predictor of *C. nodosa* loss over time, and with Montefalcone et al. (2008) and Carreño and Lloret (2021), who documented similar effects of boat anchoring on *P. oceanica* meadows. Previous studies conducted in the eastern Mediterranean also found that disturbances such as coastal infrastructure and sewage discharges severely affect seagrasses, by causing both physical damage and chemical alteration of waters (Brodersen et al. 2018). Interestingly, seagrasses have shown contrasting responses to anthropogenic stressors in the NW Mediterranean over the last decades: *Posidonia oceanica* experienced a marked long-term regression starting in the late 19th–early 20th century, followed by a partial stabilization in the early 2000s, whereas *C. nodosa* beds increased in extent over the same period (Burgos et al. 2017). A comprehensive risk assessment should also account for climate change related impacts. Hence, *P. oceanica* may lose around 70–75% of its suitable habitat by 2050 and could face functional extinction by 2100 under severe scenarios, while *C. nodosa* is projected to lose between ~20% and 46% of its habitat depending on the scenario (Chefaoui et al. 2018).

Highly susceptible habitats such as assemblages of dark and semi-dark marine caves, coralligenous assemblages and photophilic infralittoral algae communities on natural substrates are split between the medium-, low- and no-Risk classes: this pattern reflects their occurrence in remote locations with few or none overlapping stressors. Nonetheless, in elaborating effective and comprehensive management strategies, it should be borne in mind that macroalgal forests are extremely active in carbon storage and sequestration, and their preservation is fundamental to counteract the ongoing climate change and to preserve a high diversity of species (Duarte et al. 2022). Shellfish farms, which have moderate risk values, represent a key blue economy sector for the area, and any loss would have a direct economic impact at local and national level. Although no peer-reviewed study has addressed the negative interaction between sea breams and shellfish farms in the Gulf of La Spezia, several national newspapers reported remarkable income losses over the last 3 years among shellfish farmers, attributable to the increase in sea bream abundance and voracity (Assandri 2022; Calandri 2023). Similar patterns of sea bream predation on shellfish farms have been documented across the Mediterranean, with reports of significant economic losses in Croatia (Glamuzina et al. 2014), France, and other coastal areas (Avdelas et al. 2021). Recent studies have documented substantial predation by sea breams escaped from fish farms on shellfishes, causing notable economic losses (Lin and Han 2025).

In this study, the main port only affects the habitats located closer to the port dam, i.e. shellfish farms and *E. elongata*. Its limited impact can be explained considering both the incomplete habitat mapping within the port area and the marine currents dynamic as affected by the dam. At this regard, Ciuffardi et al. (2025) reported that the presence of the dam substantially alters local hydrodynamics, reducing current velocity by 50% and creating modified flow patterns. This directly reduces the buffer zone of this stress layer outside the dam. Previous studies (Peirano and Bianchi 1997) report that the port-related activities definitively contributed to the decline of seagrasses within the port area that occurred in the past, and it is safe to state that the strong anthropogenic impacts associated with port operations have had a role in the local biodiversity decline of the previous decades. Interestingly, the altered water circulation and the incomplete habitat mapping within the port area can also explain the absence of any ecological impact directly attributable to the finfish aquaculture plant, since these plants are known to be an important source of chemical and biological pollution in marine habitats (Carballeira Braña et al. 2021).

### Contributions of the Research Findings Beyond the Study Region

The adopted methodology allowed for the identification of critical areas where human activities have the potential to jeopardise valuable marine habitats in the test site. Its transparency, ease of replication, and clarity for stakeholders make it a sound choice to build ecosystem-based knowledge, which is essential for developing management actions and strategies aimed at balancing socio-economic activities with the preservation of biodiversity and ecosystem services (Natural Capital Project 2014). Results are extremely useful in developing issue-oriented solutions, which are crucial for the implementation of MSP at the local and regional scales, as they allow identifying and tackling the unique challenges that each location has to face (Guo et al. 2024; Zhang et al. 2022). The nature of the InVEST HRA model enables stakeholders to collaboratively explore alternative sea-use scenarios and assess potential trade-offs among marine exploitation and habitat conservation. The data availability concerning biodiversity, human activities, and habitats response to stressors, makes the present approach particularly feasible for ERA studies in the northern Mediterranean area; like many other models of the InVEST suites (Yang et al. 2020; Benra et al. 2021; Gashaw et al. 2022), it could also be adapted to data scarce regions.

The production of spatially explicit data is essential to support the creation of decision support tools that organize, analyse, and inform the MSP process by modelling and projecting future scenarios (Stamoulis et al. 2015). A critical methodological development could be represented by the integration of the adopted methodology with direct in-real-time field measurements, such as those collected by several European research infrastructures (e.g. JERICO-RI). Recently, a new high-resolution data acquisition system has been installed in the study site (Smart Bay Santa Teresa underwater observatory, part of both e-LTER and JERICO-RI), providing a characterization of seawater changes at local scale. The acquired physical and biogeochemical data have confirmed that the gulf is suffering the effects of global warming, with an increase in sea temperature (0.045 °C ± 0.003 °C per year) and frequency of Marine Heat Waves from 2021 to 2024 (Ciuffardi et al. 2025). Together with human impacts, these changes have the potential to severely jeopardise ecological functions, compromising the ability of marine habitats to provide ESs, and eventually hindering the sustainable coastal development (O’Hara et al. 2021). Where available, the integration of model’s outputs with continuous physical and biogeochemical data, such as these collected within the Smart Bay Santa Teresa framework (Ciuffardi et al. 2025), may provide a more comprehensive risk assessment of the area, allowing for monitoring parameters connected to the ecosystem’s health, such as water temperature, salinity, pH and dissolved O_2_ and CO_2_. Such integration could favour the inclusion of climate-change related environmental modifications, which exert direct negative impact on Mediterranean biological and functional diversity (Lacoue-Labarthe et al. 2016) into the model. For example, water heating and acidification could increase the score of consequence criteria embedded into the model, e.g by enhancing the natural mortality, reducing the recruitment rate or exacerbating the change in structure, thus making habitats more susceptible to stressors. Therefore, the methodology herein adopted provides a standardized and transferable tool that can support the implementation of the EU Maritime Spatial Planning Directive, the EU Biodiversity Strategy 2030, and regional frameworks such as the UNEP/MAP Barcelona Convention.

The use of the InVEST HRA model poses some limitations. First, as with any expert-based assessment, results may be influenced by individual cognitive biases, generated from knowledge of specific case studies or personal experience (Hulme et al. 2012). Secondly, the analysis does not consider the impact of past human activities. Data availability was also a limitation: the main port area—once hosting valuable habitats prior to the intensification of harbour activities—lacks habitat mapping data, resulting in an incomplete assessment. Such an aspect should be considered before operationalizing the model’s outputs, as results might neglect important habitats located inside the port area. Additionally, the model accounts only for direct impacts of stressors on habitats and does not include any potential indirect interaction that might take place. Despite using data collected at the local scale, which should limit uncertainties, a quantitative uncertainty assessment is also lacking, and might be conducted in future studies, with the progression of seabed samplings in the Gulf of La Spezia. Due to the limitations of the InVEST HRA model, which include the nature of the scoring process and the relative scale of the outcome, risk calculations shall not be compared from those obtained in other studies (Natural Capital Project 2014).

## Conclusions

This study tested, for the first time in the Mediterranean Sea, the InVEST Habitat Risk Assessment model. This Environmental Risk Assessment approach provided a comprehensive spatial framework to understand habitat vulnerabilities and limitations in a representative test site (La Spezia Gulf) to enhance Maritime Spatial Planning.

For the case study, the main findings were that:

1. Risk is concentrated within the first 250 metres offshore from the coast and around islands, reflecting the spatial overlap between susceptible habitats and human activities.
2. Seagrass habitats - particularly *P. oceanica* formations and *C. nodosa* beds - face the highest cumulative risk due to their high sensitivity and exposure to multiple anthropogenic stressors.
3. The major stressors are activities/infrastructures like leisure boat anchorage areas, sewage pipelines, touristic ports, and nautical facilities.

The proposed management scenario demonstrates strong potential for risk mitigation, with mean risk reductions ranging from 2.7% to 73.4%, although benefits vary significantly among habitats. Strategic actions can substantially reduce habitat risk notably, in some cases (e.g., legislation prohibiting illegal leisure boat anchoring) they already exist, though enforcement remains inadequate.

Future management efforts should adopt an integrated approach that addresses multiple stressors simultaneously, as results indicate that coordinated actions could provide synergistic benefits across the entire marine ecosystem, thus enhancing the provision of critical ESs.

The methodological framework adopted in this study, and particularly the simulation of the consequences of different management actions, has clear relevance in the Mediterranean basin (Micheli et al. 2013), where the millennial history of human coastal development insists on a unique biodiversity hotpot. Many coastal areas in the Basin are characterized by similar ecological features and face comparable pressures from maritime traffic, coastal development, and infrastructure expansion. The approach integrates multiple stressors and habitat-specific sensitivities, which should be assessed case-by-case, using locally available datasets. This approach can support the implementation of the EU Maritime Spatial Planning Directive, the EU Biodiversity Strategy 2030, and regional frameworks such as the UNEP/MAP Barcelona Convention in the Mediterranean area.

## Supplementary information

Supplementary information

## Data Availability

All data generated or analysed during this study are included in this published article (maps, tables and figures).
